# Photosynthetic and Growth Responses in a Pioneer Tree (Japanese White Birch) and Competitive Perennial Weeds (*Eupatorium* sp.) Grown Under Different Regimes With Limited Water Supply to Waterlogging

**DOI:** 10.3389/fpls.2022.835068

**Published:** 2022-03-08

**Authors:** Mitsutoshi Kitao, Hisanori Harayama, Kenichi Yazaki, Hiroyuki Tobita, Evgenios Agathokleous, Naoyuki Furuya, Toru Hashimoto

**Affiliations:** ^1^Hokkaido Research Center, Forestry and Forest Products Research Institute, Sapporo, Japan; ^2^Department of Plant Ecology, Forestry and Forest Products Research Institute, Tsukuba, Japan; ^3^Department of Ecology, School of Applied Meteorology, Nanjing University of Information Science & Technology (NUIST), Nanjing, China

**Keywords:** birch regeneration, drought, photoinhibition, Rubisco carboxylation, waterlogging

## Abstract

For a successful natural regeneration of Japanese white birch (*Betula platyphylla* var. *japonica*), competitive vegetation should be managed. Here, we clarified how soil water condition modifies the competitiveness of Japanese white birch against perennial weeds, *Eupatorium* species, based on an ecophysiological approach combining a glasshouse experiment and a field survey. We investigated photosynthetic and growth responses to various water regimes from water deficit to waterlogging (two times-a-week irrigation, three times-a-week irrigation, half waterlogging, and full waterlogging) in pot-grown seedlings of Japanese white birch and the competitive weed *Eupatorium makinoi*. The ratio of seedling height of Japanese white birch to seedling height of *E. makinoi* showed a decreasing trend from two times-a-week irrigation to full waterlogging, which suggests a lower competitiveness for light resource in Japanese white birch with increasing soil wetness. The maximum rate of Rubisco carboxylation (V_c,max_) based on unit N was lower in waterlogging treatments than in two times- and three times-a-week irrigation in Japanese white birch, whereas *E. makinoi* showed the opposite response. This suggests that N partitioning into Rubisco and/or Rubisco activation might be suppressed in Japanese white birch but enhanced in *E. makinoi* under waterlogging. The maximum photochemical efficiency of photosystem II (F_v_/F_m_) was also lower in seedlings of Japanese white birch grown under waterlogging treatments. We further conducted a field survey on the relationship between F_v_/F_m_ and topographic wetness index (TWI) in seedlings of Japanese white birch and *E. glehnii* (closely related to *E. makinoi*) naturally grown in a study site 5 years after canopy tree cutting. Lower F_v_/F_m_ was observed in seedlings of Japanese white birch with increasing TWI, whereas no significant trend was observed in *E. glehnii*, in agreement with the glasshouse experiment. Thus, keeping soils not always humid might be favorable to photosynthetic performance and growth competitive ability of Japanese white birch against *Eupatorium* species.

## Introduction

Japanese white birch (*Betula platyphylla* var. *japonica*) is a pioneer deciduous broadleaf tree species native to northern Japan, which naturally regenerates from anemochory seeds preferably in open habitats. Within the framework of low-cost forest management, natural regeneration of valuable tree species after a canopy tree cutting is expected as an alternative practice to seedling planting (Kitao et al., 2018, 2019). Accordingly, natural regeneration of Japanese white birch is expected as a low-cost afforestation, as the value of woody products of Japanese white birch is increasing. The most critical issue in the natural regeneration of Japanese white birch in northern Japan is the competition with other types of vegetation such as bonesets (*Eupatorium makinoi*, and *E. glehnii*), Sakhalin raspberry (*Rubus matsumuranus*), and sasa bamboo (e.g., *Sasa senanensis*).

Sasa bamboo is a major competitor against birch regeneration, since sasa bamboo quickly occupies an open site with rhizomes, which creates a dense vegetation cover and a thick litter layer, which prevents germination and growth of light-demanding species such as Japanese white birch (Koike, 1988; Koike et al., 2001; Doležal et al., 2009; Seiwa et al., 2009). Therefore, soil scarification is expected to be an empirical treatment to promote Japanese white birch regeneration by removing rhizomes of sasa bamboo (Yamazaki and Yoshida, 2018, 2020). However, even when rhizomes of sasa bamboo are successfully removed by scarification, other weed species would outcompete regenerating seedlings of Japanese white birch.

*Eupatorium makinoi* and *E. glehnii* are often found in open habitats after tree cutting in northern Japan. They are perennial hemicryptophytes with winter buds forming at the soil surface. They have morphologically quite similar leaves, whereas *E. makinoi* has opposite decussate leaves and *E. glehnii* has verticillated 3 to 4 leaves. As they often occur sympatrically and even undergo hybridization (Kawahara et al., 1989; Watanabe et al., 1998), their physiological traits and suitable habitats are considered similar. Besides sasa bamboo, these *Eupatorium* species can outcompete regenerating seedlings of Japanese white birch. Although previous studies investigated photosynthetic properties in Japanese white birch in relation to water, light, and CO_2_ (Koike, 1988; Koike et al., 2003; Kitao et al., 2005, 2007) and in *E. makinoi* in relation to virus infection (Funayama et al., 2001; Funayama-Noguchi and Terashima, 2006), no comparison of growth and photosynthetic characteristics between these species has been conducted to date.

Japanese white birch is a typical light-demanding species based on its leaf structure and photosynthetic traits (Koike, 1988; Kitao et al., 2000). *E. makinoi* and *E. glehnii* are found in open habitats and rarely observed in deep-shaded forest understory (Watanabe et al., 1998), which suggests that they are also light-demanding with low-shade tolerance. Accordingly, height growth is a major factor to determine intraspecific competitiveness, as light resource is essential for these light-demanding species (Kunstler et al., 2016).

Soil scarification suppresses the growth of sasa bamboo by removing its rhizomes (Yamazaki and Yoshida, 2018, 2020). Conversely, it can also modify growth conditions regarding soil water content; for example, the removal of surface soil might increase water maintenance capacity with subsoils, changes in microtopography, mounds drain water well, and pits prevent water drainage. Moderate water-deficit stress reduces photosynthetic carbon gain mainly due to a decrease in intercellular CO_2_ concentrations through stomatal closure (Medrano et al., 2002; Bota et al., 2004; Kitao et al., 2021a). Conversely, excessive water stress, that is, waterlogging causes root injury due to low levels of oxygen, which leads to the decreases in plant growth (Sairam et al., 2008; Fujita et al., 2020). The responses to drought (Kitao et al., 2006; Valladares and Sánchez-Gómez, 2006; Hallik et al., 2009; Yazaki et al., 2015; Harayama et al., 2019) and flooding tolerance (Glenz et al., 2006; Niinemets and Valladares, 2006; Poorter and Markesteijn, 2008), however, are species-specific.

Japanese white birch has a higher ability to adapt soil water deficit based on growth and photosynthetic responses to different soil water contents, compared with two other birch species native to northern Japan, *viz. B. ermanii* and *B. maximowicziana* (Koike et al., 2003). Conversely, Japanese white birch might be sensitive to flooding, as is indicated by the greater sensitivity of growth and photosynthesis to flooding in birches compared with flooding-tolerant alders (Terazawa and Kikuzawa, 1994; Graves et al., 2002; Iwanaga and Yamamoto, 2008). Although growth and photosynthetic responses to soil water conditions in *Eupatorium* species have not been investigated, *E. makinoi* and *E. glehnii* might be tolerant to waterlogging since both species were found in a wetland (Shizukari Mire, Hokkaido, Japan) (Ahyoung et al., 2016).

As the first step toward promoting regeneration of Japanese white birch, we should clarify preferable soil water conditions for growth and photosynthetic traits of Japanese white birch and possible competitor *Eupatorium* species. We hypothesized that growth and photosynthetic rate of Japanese white birch might decrease under wet soil conditions such as waterlogging, whereas those of *Eupatorium* species might not be affected under the same conditions. To test the hypothesis, we conducted (1) a glasshouse experiment to study growth (including height growth) and photosynthetic responses in seedlings of Japanese white birch and plants of *E. makinoi* grown under various water conditions, from limited water supply to full waterlogging, and (2) a field survey of photosynthetic activity, indicated by a chlorophyll fluorescence coefficient, F_v_/F_m_ (maximum photochemical efficiency of photosystem II), in Japanese white birch seedlings and coexisting *E. glehnii* in relation to various soil water conditions in an open site with and without soil scarification after cutting canopy trees of Sakhalin fir (*Abies sachalinensis*). In this study, we employed an ecophysiological approach combining a glasshouse experiment and a field survey to clarify how soil water condition influences the competitiveness of Japanese white birch against perennial weeds, *Eupatorium* species.

## Materials and Methods

### Growth and Photosynthetic Properties in Seedlings of Japanese White Birch and *Eupatorium makinoi* Grown Under Different Water Regimes

#### Plant Materials

Seeds of Japanese white birch and *E. makinoi* were sown in plastic pots (5.5 cm in diameter, 13 cm in height, and 200 ml in volume) in June 2020. Commercial seeds of Japanese white birch were harvested in Sapporo, Japan in 2018. Seeds of *E. makinoi* were collected by these authors at the experimental forest of Hokkaido Research Center, FFPRI (43°N, 141°E; 180 m a.s.l.), in autumn, 2019. All seeds were kept in a refrigerator (4°C) until sowing in June 2020. Germinated seedlings of both species were grown with adequate fertilization in a glasshouse at Hokkaido Research Center. Totally, 2 g of slow-released fertilizer (Osmocote Exact Standard 15-9-11 + TE, HYPONeX Japan, Osaka, Japan), corresponding to 300 mg N pot^–1^, was given. Adequate water was supplied with sprinklers on a daily basis.

Four water regimes were applied from the end of July to the end of September 2020; (1) limited water supply (two times-a-week irrigation: totally ≈ 140 ml pot^–1^ week^–1^), (2) adequate water supply (three times-a-week irrigation: totally ≈ 210 ml pot^–1^ week^–1^), (3) half waterlogging (always wet soils: pots were half soaked up to ≈ 7 cm in height from the bottom), and (4) full waterlogging (pots were soaked into water up to soil surface). A total of six seedlings of Japanese white birch and six seedlings of *E. makinoi* were randomly placed into a meshed tray of freely drainage for two times- and three times-a-week irrigation treatments. Similarly, six seedlings of Japanese white birch and six seedlings of *E. makinoi* were grown in a tray with tapped water for half and full waterlogging treatments. There were four tray replications of each water regime. Soil volumetric water content (SWC) in two times- and three times-a-week irrigation was monitored with soil moisture smart sensors (EC5, Onset Computer Corporation, Bourne, MA, United States) combined with data loggers (H21-USB, Onset Computer Corporation). SWC just after irrigation was around 26% in both treatments, whereas it decreased to 10.6 and 14.3% just before irrigation in two times- and three times-a-week irrigation treatments. These SWC values represent averages for each water regime, as measured by four sensors (2 × Japanese white birch and 2 × *E. makinoi*).

#### Measurements of Gas Exchange and Chlorophyll Fluorescence

Light-saturated net photosynthetic rate (A), stomatal conductance (g_s_), intercellular CO_2_ concentration (C_i_), and chlorophyll fluorescence parameters were measured in fully expanded mature leaves of Japanese white birch and *E. makinoi* with a portable photosynthesis system (LI-6800, Li-Cor, Lincoln, NE, United States) combined with a leaf chamber fluorometer (LI-6800-01A, Li-Cor). Measurements were taken at an ambient CO_2_ concentration of 400 μmol mol^–1^, a light intensity of 1,000 μmol m^–2^ s^–1^, a leaf temperature of 25°C, and a relative humidity of ≈ 70%. To measure the capacity of photosynthesis, plants grown under two times- and three times-a-week irrigation were fully irrigated in the evening of the day before the measurements. Based on A and C_i_, maximum rate of Rubisco carboxylation (V_c,max_) was estimated with the “one-point method” (de Kauwe et al., 2016), using Rubisco kinetic parameters at a leaf temperature of 25°C (Bernacchi et al., 2001).

Regarding chlorophyll fluorescence parameters, maximum and minimum fluorescence under dark (F_m_ and F_0_, respectively) were determined for overnight dark-adapted leaves. After the leaves reached a photosynthetic steady state under saturating light (1,000 μmol m^–2^ s^–1^), steady-state fluorescence (F_s_) and maximum and minimum fluorescence under light (F_m_’ and F_0_’) were determined. Maximum photochemical efficiency of photosystem II [F_v_/F_m_, = (F_m_-F_0_)/F_m_] and quantum yield of photosystem II electron transport [YII, = (F_m_’-F_s_)/F_m_’] were calculated (Genty et al., 1989; Hendrickson et al., 2004; Klughammer and Schreiber, 2008). Leaf absorptance (ABS) was measured with a leaf spectrometer (CI-710, CID Bio-Science, Camas, WA, United States). Electron transport rate (ETR) was calculated as ETR = ABS × light intensity × YII × 0.5 (Demmig-Adams et al., 1996).

Leaves used for the gas exchange measurements were sampled. Then, leaf area was determined using a computer-connected scanner (LiDE210, Canon, Tokyo, Japan) and an image analysis software (LIA32 version 0.3781).^1^ Leaves were dried to constant weight at 70°C. Dry mass-based leaf nitrogen content (N_mass_) was determined by an analysis system composed of an N/C determination unit (SUMIGRAPH, NC 800, Sumika Chem. Anal. Service, Osaka, Japan), a gas chromatographer (GC 8A, Shimadzu, Kyoto, Japan), and a data processor (Chromatopac, C R6A, Shimadzu). N_mass_ was converted to area-based leaf N content (N_area_) by multiplying with leaf mass per area (LMA). N-based V_c,max_ was also calculated as area-based V_c,max_ divided by N_area_.

#### Growth and Biomass Allocation

Plants were harvested at the end of September 2020, separated into shoots (leaves + stems), and roots, and dried at 70°C to constant weight.

#### Statistical Analysis for Glasshouse Experiment

An analysis of variance (ANOVA) was applied to investigate the effects of water regimes on photosynthetic traits in seedlings of Japanese white birch and *E. makinoi*. One representative seedling per tray was selected for each species, giving a total of four real replicates per treatment per species. Similarly, the effect of water regimes on the ratio of seedling height, and total biomass of Japanese white birch to *E. makinoi*, which was calculated for each tray, was analyzed with an ANOVA. A generalized linear mixed model was applied with an assumption of log-gamma distribution to analyze growth properties (seedling height, total biomass, S:R ratio, and root biomass); water regime was a fixed factor and tray a random factor. We used the glmer function of the R package lme4 for the model fitting (Bates et al., 2015) and the ANOVA function of the R package car for the analysis of the deviance table (Fox and Weisberg, 2019). When there was at least one significant difference among water regimes based on the ANOVA, Tukey–Kramer *post-hoc* test followed. The level of significance was α = 0.05.

### Field Survey on F_v_/F_m_ in Japanese White Birch in Relation to Soil Water Condition

#### Study Site

**FIGURE 1:**
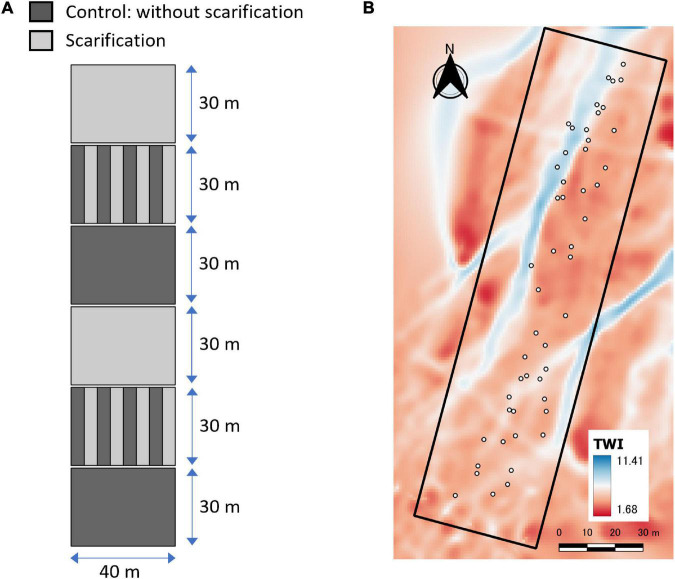
Site design for scarification **(A)** and TWI distribution with 1 m meshes **(B)** at Ikutora study site. White circles in the right panel indicate the points where the seedlings of Japanese white birch were grown for chlorophyll fluorescence measurements. The study site was located on the north-northeast slope with an inclination angle of 12°(downward from the bottom left to the top right).

Ikutora study site was established in 63-year-old forest plantations of Sakhalin fir, managed by the Kamikawa Nanbu regional forest office of the Forestry Agency, Japan, located at Ikutora, Minami Furano, Hokkaido, Japan (43.1843°N, 142.6003°E, 480–410 m a.s.l.). Details about the study site are presented in previous publications (Itô et al., 2019). The study site was located on the north-northeast slope (downslope: 185 m × lateral slope: 40 m), with an inclination angle of 12° (Figures 1A,B). Canopy tree cuttings and soil scarification were conducted in July 2015. The study site was divided into six plots: control (without scarification) × 2, full scarification × 2, and striped scarification × 2. In the striped scarification, control and scarification rows alternated at 5-m intervals (Figure 1A). In this study, the scarification procedure was conducted as “screening,” in which understory plants including sasa bamboo were picked out, and soil was shaken off to the site (Yamazaki and Yoshida, 2018). Topographic wetness index (TWI) (Beven and Kirkby, 1979) was calculated with 1-m meshes based on the topographic data obtained by an unmanned aerial vehicle (UAV) [Phantom 4 PRO(P4P), DJI, Shenzhen, China] in the first winter, 2016, after the canopy tree cutting (Figure 1B). Multiple flow algorithm was used to calculate the contributing upslope area with flow convergence of 1.1 using SAGA GIS tools integrated with QGIS (Qgis Development Team, and Qgis.org., 2021). Local slope gradient was calculated with the Zevenbergen and Thorne formula using Geospatial Data Abstraction Software Library (GDAL) in QGIS.

#### Measurements of Photosynthetic Activity Indicated by Chlorophyll Fluorescence

**FIGURE 2:**
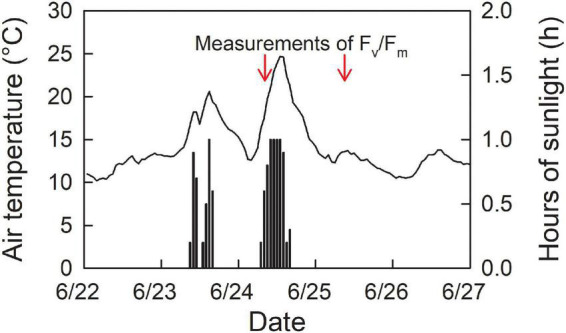
Air temperature (solid line) and hours of sunlight (bars) at Ikutora study site. Arrows indicate the timing of overnight dark-adapted F_v_/F_m_. Data were recorded at Ikutora weather station (3 km southwest to the site) (https://www.data.jma.go.jp/obd/stats/etrn/index.php).

Field measurements of chlorophyll fluorescence were conducted with a portable chlorophyll fluorometer (Mini-PAM II, Walz, Effeltrich, Germany) on June 24 and 25, 2020. We measured chlorophyll fluorescence in plants grown in the middle part of the study site, which includes 48 subplots (totally, *n* = 24 for control, and *n* = 24 for scarification), along the slope with a 20 m width (cf. Figure 1B). First, we chose a seedling of Japanese white birch from each subplot (Figure 1B). Then, we chose one predominant competitive weed plant (*E. glehnii* or *Sasa senanensis*, depending on the subplot) grown adjacent to the white birch seedling, which allows to compare the photosynthetic properties in plants grown at the same environmental conditions. The seedling height of Japanese white birch ranged from 110 to 260 cm (mean height = 160 cm), and that of competitive vegetation was approximately 100 cm. F_v_/F_m_ was determined for overnight dark-adapted leaves of Japanese white birch seedlings and other weed plants with a saturation pulse (light intensity of ca. 6,000 μmol m^–2^ s^–1^). Sun-exposed leaves were selected for the measurements. Leaf clips for dark adaptation were attached to the leaves in the evening of the day before the F_v_/F_m_ measurements. We measured F_v_/F_m_ in the next morning, after overnight dark adaptation. Seedlings grown in the upper half of the slope were measured in the morning of June 24, and the rest were measured on June 25, 2020. Daytime air temperature of the day before F_v_/F_m_ measurements was considerably different. The daytime average (4:00–20:00) was 17.0 and 19.3°C for 23 and 24 (Figure 2), based on the meteorological data at Ikutora weather station (3 km southwest to the site).^2^ As F_v_/F_m_ after an overnight dark adaptation is influenced by the integrated irradiance (Werner et al., 2001), and air temperature (Krause, 1994; Kitao et al., 2018) in the previous day, daytime air temperature of the day before F_v_/F_m_ measurements was shown. We took photographs above the seedlings of Japanese white birch used for the chlorophyll fluorescence measurements by placing a digital camera (Coolpix 900, Nikon, Tokyo, Japan) combined with a fisheye lens (Fisheye Lens, FC-E8, Nikon) above the seedlings. Global site factor (GSF: the proportion of direct plus diffuse solar radiation at a given site relative to that in the open) was calculated based on the hemispheric photographs by a canopy analysis software (HemiView 2.1, Delta-T Devices Ltd., Cambridge, United Kingdom). Soil nitrogen content (SNC) was determined for undisturbed core samples (5 cm long and 100 cm^3^ in volume) derived from a soil depth of 0–30 cm, from the 48 representative points of subplots (around the center of subplot). SNC (soil dry mass-based) was determined by a CN analyzer (vario Max CN, elementar, Langenselbold, Germany).

#### Statistical Analysis for the Field Survey

Regarding the field measurements of F_v_/F_m_, a stepwise method for variable selection for multiple regression analysis was used for quantitative evaluation of the influence of the explanatory factors (initial variables: date of measurements, scarification, GSF, SNC, and TWI) on F_v_/F_m_. Stepwise regressions were used to define the subset of effects that would altogether provide the smallest corrected Akaike information criterion (AIC) in subsequent modeling. An analysis of covariance (ANCOVA) was applied to investigate the effects of fixed factors (date of measurements and scarification) and covariance (TWI) on site-related environmental traits (GSF and SNC) at Ikutora study site. The level of significance for coefficients was set at 0.05.

## Results

### Gas Exchange and Chlorophyll Fluorescence in Pot-Grown Seedlings of Japanese White Birch and *Eupatorium makinoi* Grown Under Various Water Regimes

**FIGURE 3:**
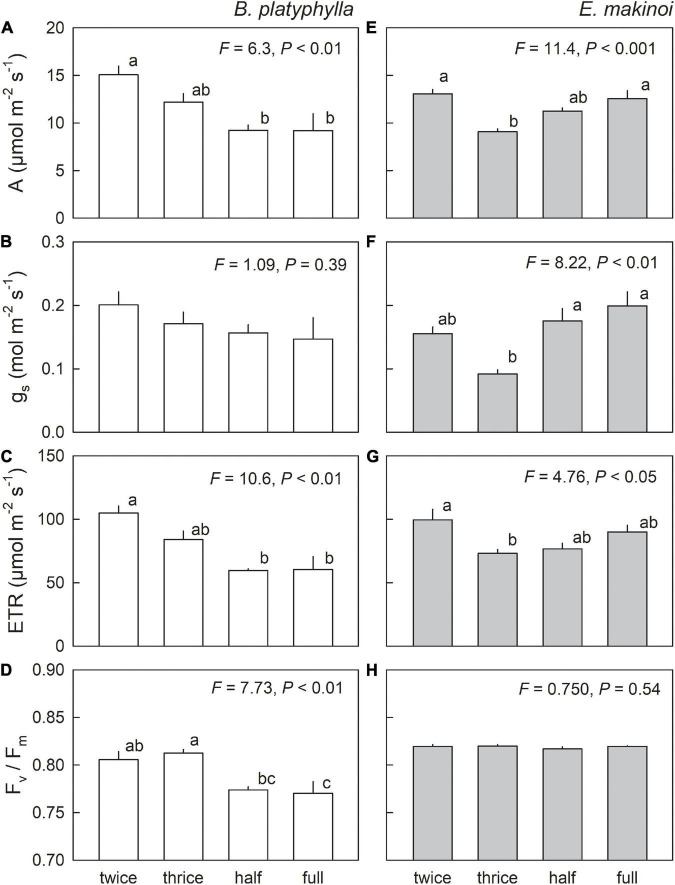
Photosynthetic traits: A **(A,E)**, g_s_ **(B,F)**, ETR **(C,G)**, and F_v_/F_m_ **(D,H)** in seedlings of Japanese white birch (white bars) and *Eupatorium makinoi* (gray bars) grown under various water regimes. Twice: two times-a-week irrigation, thrice: three times-a-week irrigation, half: half waterlogging, full: full waterlogging. To measure the capacity of photosynthesis, plants grown under two times- and three times-a-week irrigation were fully irrigated in the evening in the prior day for the measurements. Values are means + SE (*n* = 4). Different letters above means of the four experimental conditions indicate significant differences at *p* < 0.05.

Lower light-saturated net photosynthetic rate (A) was observed in seedlings of Japanese white birch grown under waterlogging treatments (half and full waterlogging) whereas stomatal conductance (g_s_) showed no significant difference among water regimes (Figures 3A,B). Conversely, the lowest A was observed in seedlings of *E. makinoi* grown under adequate irrigation (three times-a-week) and having the lowest g_s_ (Figures 3E,F). Electron transport rate (ETR), calculated from chlorophyll fluorescence and leaf absorptance, showed similar trends to A observed in both Japanese white birch and *E. makinoi* (Figures 3C,G). Lower ETR was observed in the two waterlogging treatments in Japanese white birch, whereas such a decrease was not observed in seedlings grown under the two waterlogging treatments in *E. makinoi*. Maximum photochemical efficiency of PSII (F_v_/F_m_) after one-night dark adaptation showed a decreasing trend below 0.80 in waterlogged seedlings of Japanese white birch, whereas no significant difference was observed in seedlings of *E. makinoi* (Figures 3D,H). Based on a two-way ANOVA of the effects of species, water regime, and their interaction on photosynthetic traits (Supplementary Table 1), interaction between species and water regime significantly affected (*p* < 0.05) all the photosynthetic traits described above. Thus, photosynthetic responses to various water regimes might be species-specific.

**FIGURE 4:**
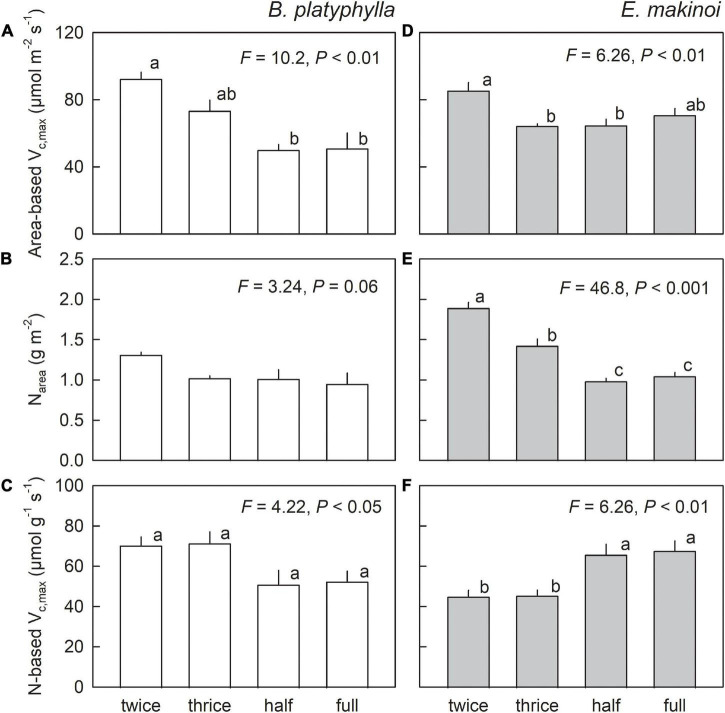
Area-based V_c,max_ **(A,D)**, area-based leaf N content (N_area_) **(B,E)**, and N-based V_c,max_ **(C,F)** in seedlings of Japanese white birch (white bars) and *Eupatorium makinoi* (gray bars) grown under various water regimes. Twice: two times-a-week irrigation, thrice: three times-a-week irrigation, half: half waterlogging, full: full waterlogging. Values are means + SE (*n* = 4). Different letters above means of the four experimental conditions indicate significant differences at *p* < 0.05.

Area-based V_c,max_ showed similar trends to A and ETR in both species (Figures 4A,D). Lower area-based V_c,max_ was observed in seedlings of Japanese white birch grown under the waterlogging treatments, whereas no such a decrease was observed in seedlings of *E. makinoi* grown under the waterlogging treatments. Area-based leaf N content (N_area_) showed no significant difference among the water treatments in Japanese white birch, whereas higher N_area_ was observed with water limitation in *E. makinoi* (Figures 4B,E). No significant *post-hoc* difference was detected in N-based V_c,max_ in seedlings of Japanese white birch among the water regimes, although water regime was overall a significant factor (Figure 4C). When data were pooled as periodic irrigation (two times- and three times-a-week irrigation) and waterlogging treatments (half and full waterlogging), a significant difference was observed between the two groups (*F* = 14.6, *p* < 0.01). Conversely, a significantly higher N-based V_c,max_ was observed in seedlings of *E. makinoi* grown under waterlogging treatments (Figure 4F). Two-way ANOVA of the effects of species, water regime, and their interaction on photosynthetic traits (Supplementary Table 1) revealed significant effect of the interaction (*p* < 0.05) on area-based V_c,max_, N_area_, and N-based V_c,max_.

### Growth and Biomass Allocation in Seedlings of Japanese White Birch and *Eupatorium makinoi* Grown Under Various Water Regimes

**FIGURE 5:**
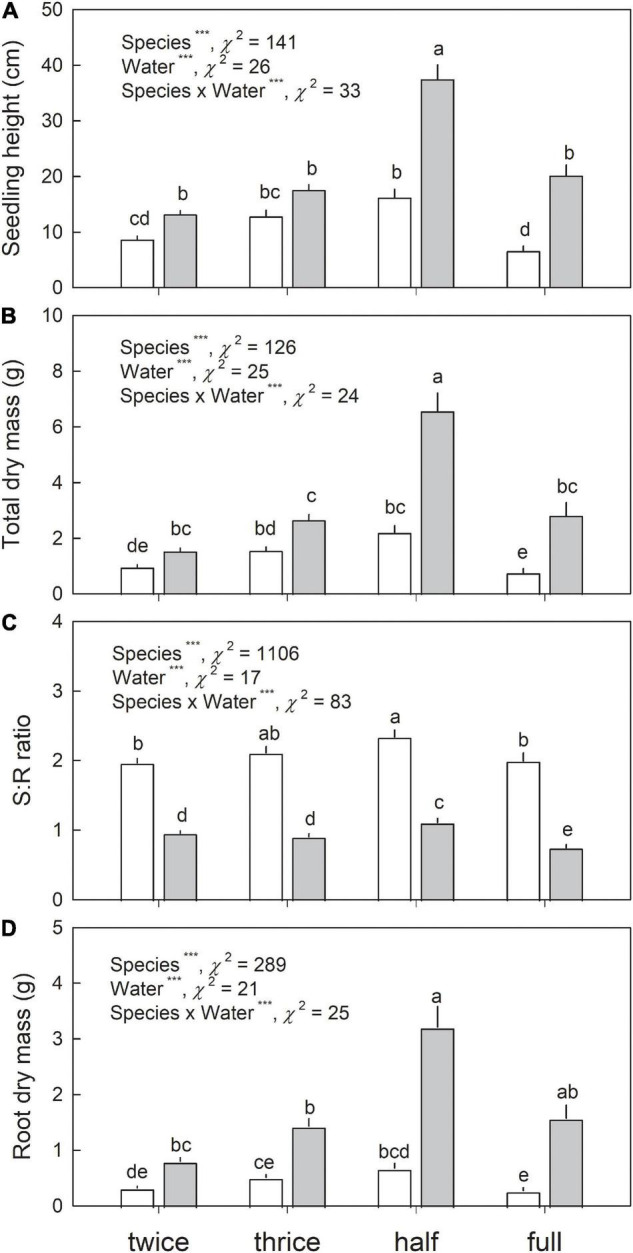
Seedling height **(A)** total biomass **(B)**, shoot: root ratio **(C)**, and root biomass **(D)** in seedlings of Japanese white birch (white bars) and *Eupatorium makinoi* (gray bars) grown under various water regimes. Twice: two times-a-week irrigation, thrice: three times-a-week irrigation, half: half waterlogging, full: full waterlogging. Values are mean + SE (*n* = 20–24). ***denotes significant main effect at *p* ≤ 0.001. Different letters above means of the eight experimental conditions indicate significant differences at *p* < 0.05.

**FIGURE 6:**
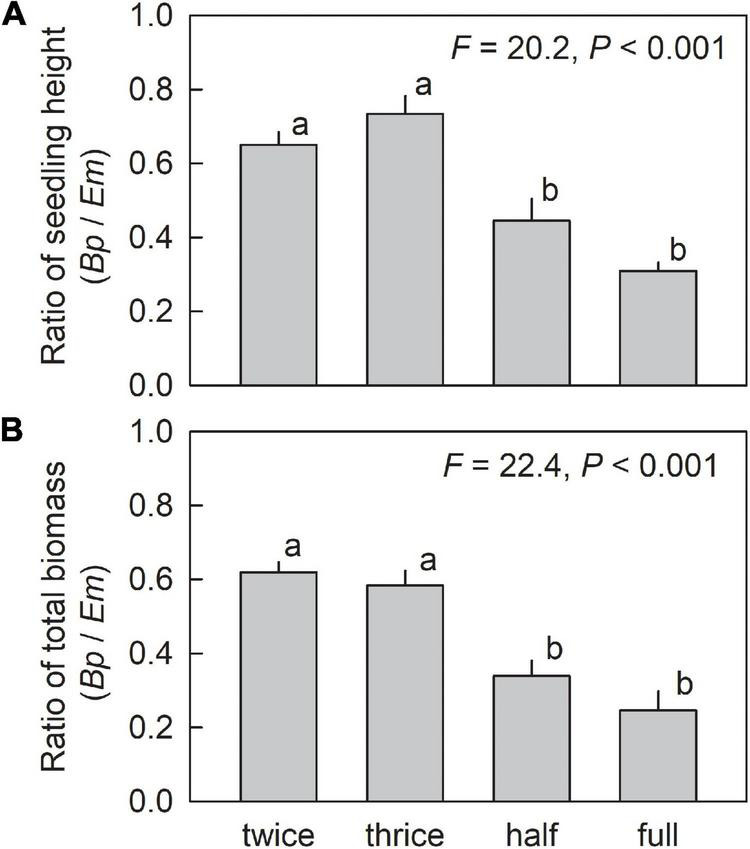
Ratio of seedling height **(A)** and total biomass **(B)** of Japanese white birch (*Bp*) to *Eupatorium makinoi* (*Em*) grown under various water regimes. Twice: two times-a-week irrigation, thrice: three times-a-week irrigation, half: half waterlogging, full: full waterlogging. To calculate the ratio, mean values of seedling height and total biomass per each tray for each species were used (*n* = 4). Values are mean + SE. Different letters above means of the four experimental conditions indicate significant differences at *p* < 0.05.

In both species, the highest growth, indicated by seedling height and total biomass, was observed in seedlings grown under half waterlogging (Figures 5A,B). In comparison with three times-a-week irrigation, which could be considered as control with adequate irrigation, significantly lower seedling height and total biomass were observed under full waterlogging in Japanese white birch. No significant difference in seedling height and total biomass was observed between three times-a-week irrigation and full waterlogging in *E. makinoi*, but significantly higher seedling height and total biomass were observed in half waterlogging treatment. Higher total biomass was generally observed in *E. makinoi* than in Japanese white birch irrespective of water regimes. Conversely, although a generally higher seedling height was observed in *E. makinoi*, no significant difference in seedling height was observed between Japanese white birch and *E. makinoi* under three times-a-week irrigation. Generally, a higher S/R ratio was observed in Japanese white birch than in *E. makinoi* (Figure 5C). In Japanese white birch, the highest S/R ratio was observed in half waterlogging treatment. Drier soil conditions resulted in lower S/R ratio, whereas full waterlogging also decreased S/R ratio compared with half waterlogging (Figure 5C). Similarly, seedlings of *E. makinoi* grown under three times- and two times-a-week irrigation, and full waterlogging showed a significantly lower S/R ratio compared with those grown under half waterlogging (Figure 5C). Generally, higher root dry mass was observed in *E. makinoi* than in Japanese white birch irrespective of water regimes (Figure 5D). In Japanese white birch, the highest root dry mass was observed in half waterlogging treatment, and significantly lower root dry mass was observed in full waterlogging treatment when compared to half waterlogging. In *E. makinoi*, the highest root dry mass was observed in half waterlogging treatment, and significantly lower rood dry mass was observed in three times- and two times-a-week treatments when compared to half waterlogging treatment. The ratio of seedling height and that of total biomass of Japanese white birch to *E. makinoi* were significantly lower in the waterlogging treatments (half and full waterlogging) than in the limited and adequate irrigation treatments (two times- and three times-a-week irrigation) (Figure 6). The ratio of seedling height of Japanese white birch to *E. makinoi* was generally higher than that of total biomass across the water treatments.

### Field Measurement of F_v_/F_m_ in Relation to Topographic Wetness Index in Seedlings of Japanese White Birch, *Eupatorium glehnii*, and *Sasa senanensis* Grown at Ikutora Study Site

**FIGURE 7:**
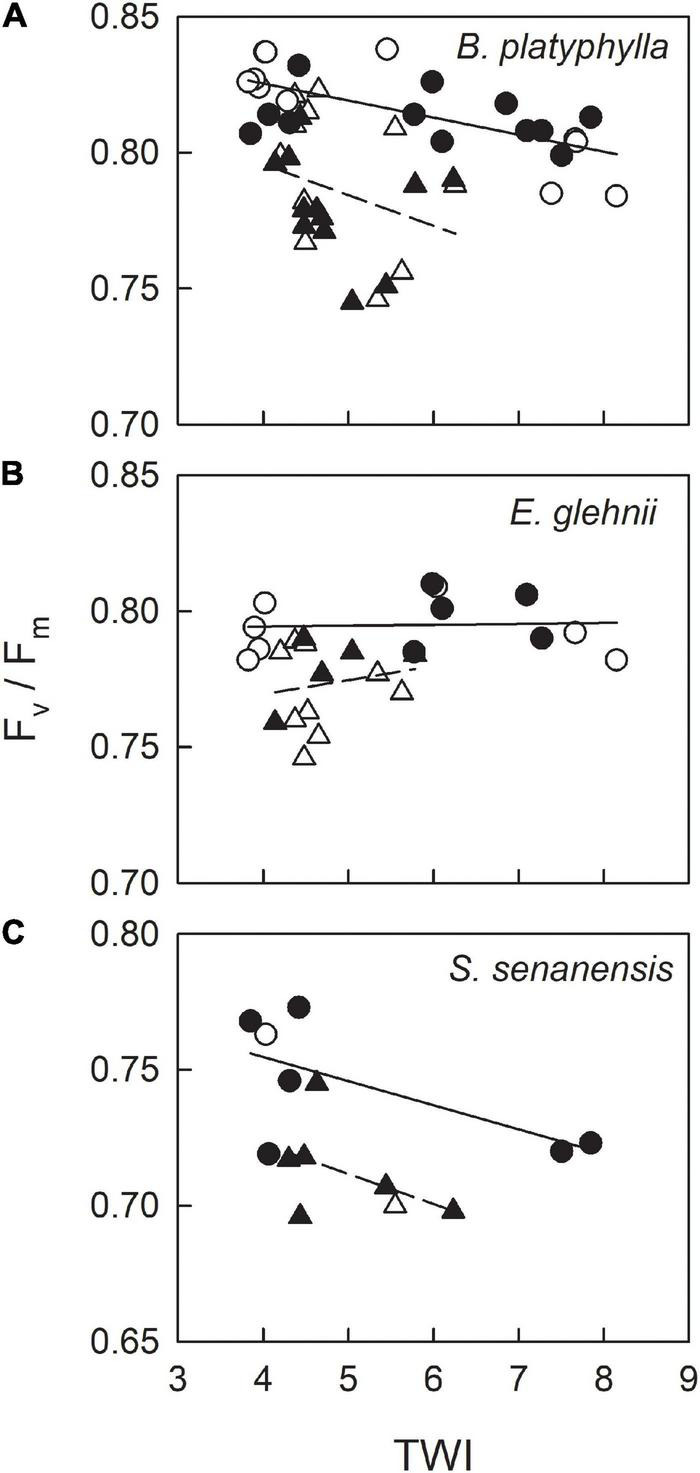
Relationship between F_v_/F_m_, measured on June 24 (triangle) and 25 (circle) and TWI in seedlings of Japanese white birch **(A)**, *Eupatorium glehnii* **(B)**, and *Sasa senanensis* **(C)** grown at Ikutora study site. Closed symbols indicate seedlings grown in the scarification plots, and open symbols indicate those in control plots without scarification. Dashed line represents linear regression for pooled data on the June 24 irrespective of scarification. Solid line represents linear regression for pooled data on the June 25, irrespective of scarification. Results of the multiple regression analyses are shown in Table 1.

**FIGURE 8:**
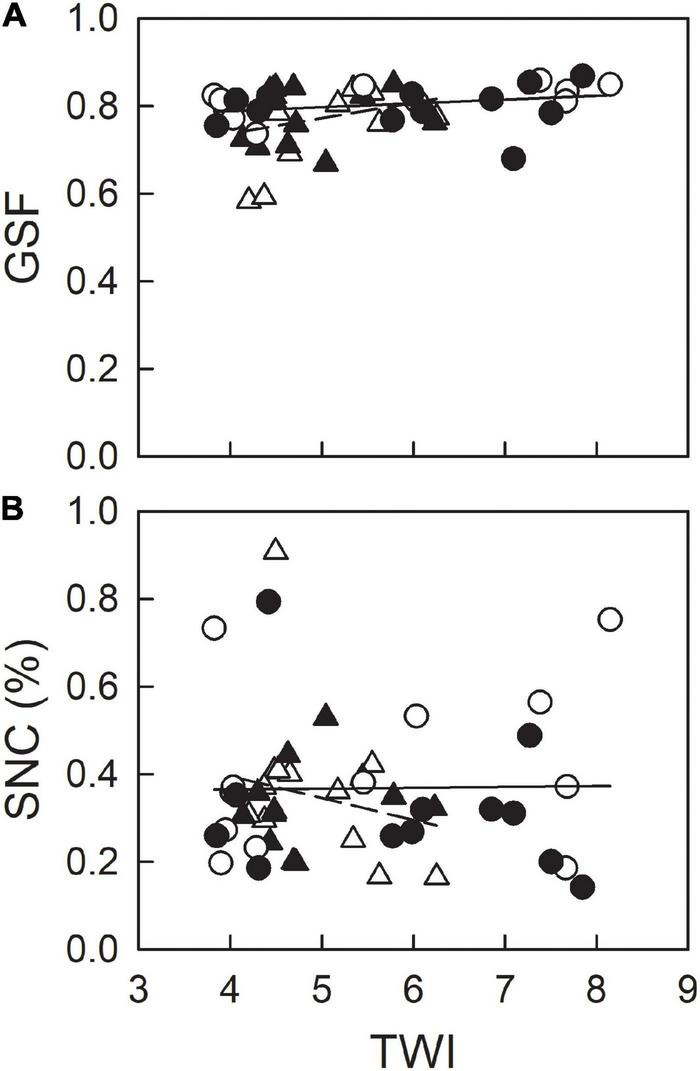
GSF **(A)** and soil N content (SNC) **(B)** as a function of TWI, with (closed) or without (open) scarification at the subplots where chlorophyll fluorescence measurements were taken on June 24 (triangle) and 25 (circle) at Ikutora study site.

Topographic wetness index (unitless) estimated with 1-m meshes ranged between 1.7 and 11.4 at Ikutora study site (Figure 1B). Seedlings of Japanese white birch, used for the measurements of F_v_/F_m_, were grown under a considerably wide range of soil water conditions (Figure 7). The values of F_v_/F_m_ on June 25 were higher than those on June 24, irrespective of species. For each date, no significant effect of scarification was observed for either species. Conversely, F_v_/F_m_ showed a decreasing trend with increasing TWI in seedlings of Japanese white birch (Figure 7A and Table 1), whereas no significant trend was observed in seedlings of *E. glehnii* and *S. senanensis* (Figures 7B,C and Table 1). It is noteworthy that a relatively lower F_v_/F_m_ was observed in *S. senanensis* among the three species. Air temperature was lower on June 23 than on June 24, the previous day before F_v_/F_m_ measurements, corresponding to the measurements on June 24 and 25, respectively (Figure 2). As the date of measurements (date: June 24 and 25) corresponded to the upper and lower half of the slope at Ikutora study site, respectively, no significant effects of date on GSF and SNC were observed, which suggests no significant difference in GSF and SNC between upper and lower half of the slope (Figure 8 and Table 2). Furthermore, no significant effects of scarification and TWI were observed on GSF and SNC (Figure 8 and Table 2).

**TABLE 1:** Summary of multiple linear regression of F_v_/F_m_ in seedlings of Japanese white birch (*Betula platyphylla*), *Eupatorium glehnii*, and *Sasa senanensis* grown at Ikutora study site.

	Summary measures		Regression coefficients				
Species	*R* ^2^	*P*	Intercept	Variable	Coefficients	*P*	VIF
*B. platyphylla*	0.46	<0.001	0.820	TWI**	–0.00698	<0.01	1.12
	(*n* = 46)			Date***	0.0347	<0.001	1.12
*E. glehnii*	0.435	<0.001	0.771	Date***	0.0212	<0.001	1.00
	(*n* = 26)			Scar^ns^	0.00738	0.16	1.00
*S. senanensis*	0.69	<0.01	0.851	TWI^ns^	–0.00460	0.24	1.32
	(*n* = 14)			Date**	0.0382	<0.01	1.05
				GSF^ns^	–0.174	0.10	1.20
				SNC^ns^	0.0496	0.14	1.17

*Initial explanatory variables affecting F_v_/F_m_: TWI, date of measurements (Date), scarification (Scar), GSF, and SNC. Date and Scar have the value of 0 or 1, where date = 0: June 24, and 1: June 25, Scar = 0: control (without scarification), and 1: scarification, respectively. Stepwise regressions were undertaken to define the subset of effects that would altogether provide the smallest corrected AIC in subsequent modeling. As a measure of multicollinearity, variance inflation factor (VIF) is demonstrated.*

**denotes significant difference in the coefficients at p ≤ 0.01, and ***p ≤ 0.001. ns indicates no significant difference.

**TABLE 2:** F-statistics of GSF and SNC at Ikutora study site.

	F-statistics (*F*_1,43_)			
	Date	Scar	Date x Scar	TWI
GSF	1.71^ns^	0.00 ^ns^	1.15^ns^	2.99^ns^
SNC	0.15^ns^	1.70^ns^	0.21^ns^	0.01^ns^

*ANCOVA was conducted with measurement date (Date), which corresponds to different site locations: upper slope (first day) and lower slope (second day) at Ikutora site, respectively, and scarification (Scar) as fixed factors, and with TWI as a covariance. ns indicates that the factor is non-significant (p > 0.05). Refer to GSF and SNC as a function of TWI (cf. Figure 8).*

## Discussion

### Photosynthesis and Growth Responses in Pot-Grown Seedlings of Japanese White Birch and *Eupatorium makinoi* Grown Under Various Water Regimes

Regarding competition between light-demanding pioneer Japanese white birch and perennial hemicryptophyte *E. makinoi*, shoot height growth is most relevant for light acquisition and might be a determinant factor achieving competitive success (Koike, 1988; Kitao et al., 2000). As seedlings of Japanese white birch and *E. makinoi* were grown in separate pots, below-ground competition and mutual shading were not at play. We assessed the competition advantage regarding height growth when no interspecific competition occurs (Kunstler et al., 2016). The relatively greater height growth in *E. makinoi* under wet soil conditions might further suppress the growth of Japanese white birch *via* shading when competition occurs. Compared with Japanese white birch, *E. makinoi* had about half S/R ratio, which suggests a greater amount of carbohydrate allocation into the root system. Japanese white birch preferably allocates biomass into shoot (Kitao et al., 2015, 2021b; Tobita et al., 2019), which might contribute to height growth, while compensating lower total biomass compared to *E. makinoi* across the water treatments.

In this study, we focused on shoot growth of the two species in the first year, following germination from seeds to simulate seed dispersal by wind in open habitats after canopy tree removal. In the second growing season, Japanese white birch continues the growth of the shoots formed in the previous year, whereas *E. makinoi* restarts shoot growth from the ground. Based on the height data, three times-a-week irrigation might keep competitiveness for light resource in Japanese white birch against *E. makinoi* in the first year. Two times-a-week irrigation might also keep the competitiveness to a certain extent, compared with half and full waterlogging. As initial growth of shoot in *E. makinoi* in the second year should be dependent on the amount of carbohydrate and nitrogen stored in the root system (Cyr and Bewley, 1989; Cyr et al., 1990), relatively drier soil conditions allow Japanese white birch to better competitiveness for height growth while suppressing root growth of *E. makinoi*, which might be advantageous for regeneration of Japanese white birch in the second growing season. Further investigation should be conducted regarding different life-history strategies [the trait dissimilarity, cf. (Kunstler et al., 2016)] of these species.

Based on the photosynthetic responses to various water regimes, Japanese white birch was suggested to be sensitive to wet soil conditions, whereas *E. makinoi* might be tolerant (Graves et al., 2002; Ferner et al., 2012; Kreuzwieser and Rennenberg, 2014). In Japanese white birch, N-based V_c,max_ significantly decreased under the two waterlogging treatments, which suggests decreased N allocation into Rubisco (Kitaoka and Koike, 2004) and/or decreased Rubisco activation (Hemantaranjan, 2014). Higher area-based V_c,max_, often observed in leaves acclimated to long-term drought, can compensate the decline in photosynthetic rate at lower intercellular CO_2_ concentration *via* stomatal closure under water-deficit stress (Panković et al., 1999; Kitao et al., 2003, 2007; Kitao and Lei, 2007). Such an acclimation might occur in seedlings of Japanese white birch grown under a limited water supply. Conversely, a decrease in N-based V_c,max_ in seedlings of Japanese white birch grown under waterlogging treatments might be the main cause of declined A, and ETR (Graves et al., 2002), consequently leading to chronic photoinhibition (a decline in overnight dark-adapted F_v_/F_m_) (Werner et al., 2002). Root injury due to low level of oxygen under waterlogging may decrease nutrient uptake including Mg (Smethurst et al., 2005), which possibly leads to a decrease in Rubisco activation *via* suppressed Rubisco carbamylation (Salvucci and Ogren, 1996; Stec, 2012).

In this study, we investigated photosynthetic capacity. A in seedlings grown under two times- and three times-a-week regimes was measured under well-irrigated condition (adequate water supply in the previous day). Therefore, A in seedlings under two times- and three times-a-week irrigation should be lower when soils were dried before periodical irrigation, which might cause lower growth rate in these water regimes compared with half waterlogging treatment. Furthermore, preferable biomass allocation into shoot (the highest S/R ratio) might also contribute to the greatest growth rate in the half waterlogging seedlings of Japanese white birch (Poorter and Remkes, 1990), even though leaves were somewhat stressed (F_v_/F_m_ < 0.8) (Björkman and Demmig, 1987).

As an acclimation to water-deficit stress, higher area-based V_c,max_ was observed in seedlings of *E. makinoi* grown under limited water supply as was observed in Japanese white birch. Conversely, *E. makinoi* showed increases in N-based V_c,max_ under waterlogging treatments in contrast to Japanese white birch. Regarding acclimation to long-term waterlogging at the root level, enhanced fermentation in roots is relevant for energy supply instead of mitochondrial respiration (Kreuzwieser et al., 2009; Kreuzwieser and Rennenberg, 2014). Because of low energy efficiency of fermentation, a greater amount of carbohydrates is required to transport from leaves to roots (Kreuzwieser et al., 2009; Kreuzwieser and Rennenberg, 2014). Such an enhanced demand for carbohydrates might upregulate Rubisco, indicated by increased N-based V_c,max_ (Ainsworth and Bush, 2011), in the situation of reduced N uptake under waterlogging (Kreuzwieser et al., 2009; Hemantaranjan, 2014). Thus, *E. makinoi* can acclimate to both limited water supply *via* an increase in N_area_ and to waterlogging *via* an enhancement of N-based V_c,max_. This might result in relatively higher A and ETR in seedlings grown under limited water supply and waterlogging, compared with seedlings supplied with adequate water (three times-a-week irrigation). *E. makinoi* might have a broad ability to acclimate to drought and waterlogging stresses, supported by relatively high F_v_/F_m_ > 0.8 across various water regimes, generally observed in healthy plants (Björkman and Demmig, 1987).

### Field Survey on F_v_/F_m_ in Japanese White Birch, *Eupatorium glehnii*, and *Sasa senanensis*

Based on the field measurements of F_v_/F_m_, there was no significant effect of scarification on F_v_/F_m_, irrespective of plant species (*B. platyphylla*, *E. glehnii*, and *S. senanensis*), which is partly supported by no effect of scarification on SNC (Figure 8B). The way of scarification “screening,” in which understory plants including sasa bamboo were picked out and soil was shaken off to the site (Yamazaki and Yoshida, 2018), might result in such a less effect of scarification on SNC and F_v_/F_m_. In all species, F_v_/F_m_ measured on June 24 showed lower values than those on June 25. As there were no locational effects on light resource, indicated by GSF and SNC (Figure 8), the lower F_v_/F_m_ on June 24 might possibly reflect an exacerbated photoinhibition due to the lower air temperature on the previous day, that is June 23 (Krause, 1994; Kitao et al., 2018).

Regarding effects of TWI on F_v_/F_m_, Japanese white birch showed decreasing F_v_/F_m_ with increasing TWI, which suggests that wet soil conditions might have an adverse effect on photosynthetic activity in seedlings of Japanese white birch (Terazawa and Kikuzawa, 1994; Graves et al., 2002). Conversely, *E. glehnii*, closely related to *E. makinoi*, showed relatively constant F_v_/F_m_ throughout the range of TWI at Ikutora study site, which suggests its acclimation ability to both dry and wet soil conditions, as is observed in the pot experiments described above. As the distribution of *Sasa palmata* (closely related to *S. senanensis*) in wetlands can be increased by a decline in the groundwater level (Umeda et al., 1988; Fujimura et al., 2013), *S. senanensis* was expected to have low ability to acclimate to wet soils and also Japanese white birch. However, no significant effect of TWI was observed in *S. senanensis*, although F_v_/F_m_ appeared to have a decreasing trend. Notably, relatively lower F_v_/F_m_ observed in *S. senanensis*, compared with the other two species, suggests that the root system of *S. senanensis* might be still immature even after 5 years of scarification.

## Conclusion

Based on a pot experiment and a field survey, wet soil conditions that include persistent moisture (half waterlogging) and complete flooding (full waterlogging) might reduce the shoot growth competitiveness in Japanese white birch against *Eupatorium* species. Thus, careful removal of rhizomes of sasa bamboo should be combined with a ground management keeping soils not always humid to preserve natural regeneration of Japanese white birch.

## Data Availability Statement

The original contributions presented in the study are included in the article/Supplementary Material, further inquiries can be directed to the corresponding author.

## Author Contributions

MK: conceptualization, investigation, formal analysis, and writing – original draft. HH, KY, NF, and TH: investigation, writing, reviewing, and editing. HT: conceptualization, writing, reviewing, and editing. EA: validation, writing, reviewing, and editing. All authors contributed to the article and approved the submitted version.

## Conflict of Interest

The authors declare that the research was conducted in the absence of any commercial or financial relationships that could be construed as a potential conflict of interest.

## Publisher’s Note

All claims expressed in this article are solely those of the authors and do not necessarily represent those of their affiliated organizations, or those of the publisher, the editors and the reviewers. Any product that may be evaluated in this article, or claim that may be made by its manufacturer, is not guaranteed or endorsed by the publisher.

## Footnotes

1
http://www.agr.nagoya-u.ac.jp/~shinkan/LIA32/

2
https://www.data.jma.go.jp/obd/stats/etrn/index.php
