# Supplementary material for: Photosynthetic and Growth Responses in a Pioneer Tree (Japanese White Birch) and Competitive Perennial Weeds (Eupatorium sp.) Grown Under Different Regimes With Limited Water Supply to Waterlogging
Source: Front Plant Sci. 2022 Mar 8;13:835068. doi: 10.3389/fpls.2022.835068 (PMC8959217; doi:10.3389/fpls.2022.835068)
Supplement: Supplementary file 1 [file Table_1.DOCX]

**Supplemental Table S1.** F-statistics of two-factorial ANOVA of the effects of species, water regime, and their interaction on photosynthetic traits in seedlings of Japanese white birch and *E. makinoi* grown under various water regimes (cf. Fig. 1 & 2). * denotes significant main effect at *P* ≤ 0.05, ** *P* ≤ 0.01, and *** *P* ≤ 0.001. ns indicates no significant main effect.

|  |  | F-statistics |  |  |
| --- | --- | --- | --- | --- |
|  |  | Species (F_1,24_) | Water (F_3,24_) | Species x Water (F_3,24_) |
| A |  | 0.01 ^ns^ | 8.12 ^***^ | 6.32 ^**^ |
| g_s_ |  | 0.93 ^ns^ | 2.30 ^ns^ | 4.63 ^*^ |
| ETR |  | 2.98 ^ns^ | 11.6 ^***^ | 4.75 ^**^ |
| F_v_/F_m_ |  | 50.2 ^***^ | 7.98 ^***^ | 6.88 ^**^ |
| Area-based V_c,max_ |  | 1.50 ^ns^ | 14.3 ^***^ | 3.89 ^*^ |
| N_area_ |  | 23.5 ^***^ | 27.6 ^***^ | 6.67 ^**^ |
| N-based V_c,max_ |  | 2.52 ^ns^ | 0.09 ^ns^ | 12.3 ^***^ |
